# Carotid Antiembolic (“Mesh”) Stents: Not Created Equal

**DOI:** 10.1016/j.jscai.2024.102429

**Published:** 2024-10-29

**Authors:** Piotr Musiałek, Lukasz Tekieli, Tomoyuki Umemoto

**Affiliations:** a Department of Cardiac and Vascular Diseases, Jagiellonian University Medical College, Krakow, Poland; b Stroke Thrombectomy-Capable Center, St. John Paul II Hospital, Krakow, Poland; c Department of Cardiovascular Medicine, Tokyo Medical and Dental University and Tokyo Institute of Science, Tokyo, Japan

**Keywords:** antiembolic stent, carotid artery stenting, stroke

Whenever feasible, strokes should be prevented.^1^ Carotid-related strokes, presenting as isolated extracranial or “tandem” (extracranial plus intracranial) lesions, account for 20% to 30% of ischemic strokes^1^; they are often large and disabling.^1^^,^^2^ Revascularization plays an important role in reducing carotid-related stroke incidence, particularly in increased-risk stenoses.^1^

Historically, carotid revascularization was best performed with surgery, but endovascular techniques have evolved significantly over the past 3 decades and are now an accepted alternative to endarterectomy.^1^ Today, neuroprotected carotid artery stenting (CAS) employing second-generation stents has outcomes similar to surgery, with some stent designs likely superior to surgery.^3^

Second-generation carotid stents (ie, antiembolic stents, dual-layer “mesh” stents) were developed to address the problem of incomplete plaque sequestration from the lumen, underlying a relative excess of 30-day minor strokes with single-layer stent CAS vs endarterectomy.^1^^,^^4^ To date, 3 carotid mesh stent designs have undergone clinical evaluation: Casper/Roadsaver (Terumo), Gore Carotid Stent (Gore), and CGuard MicroNet-covered stent (InspireMD).

**Figure 1:**
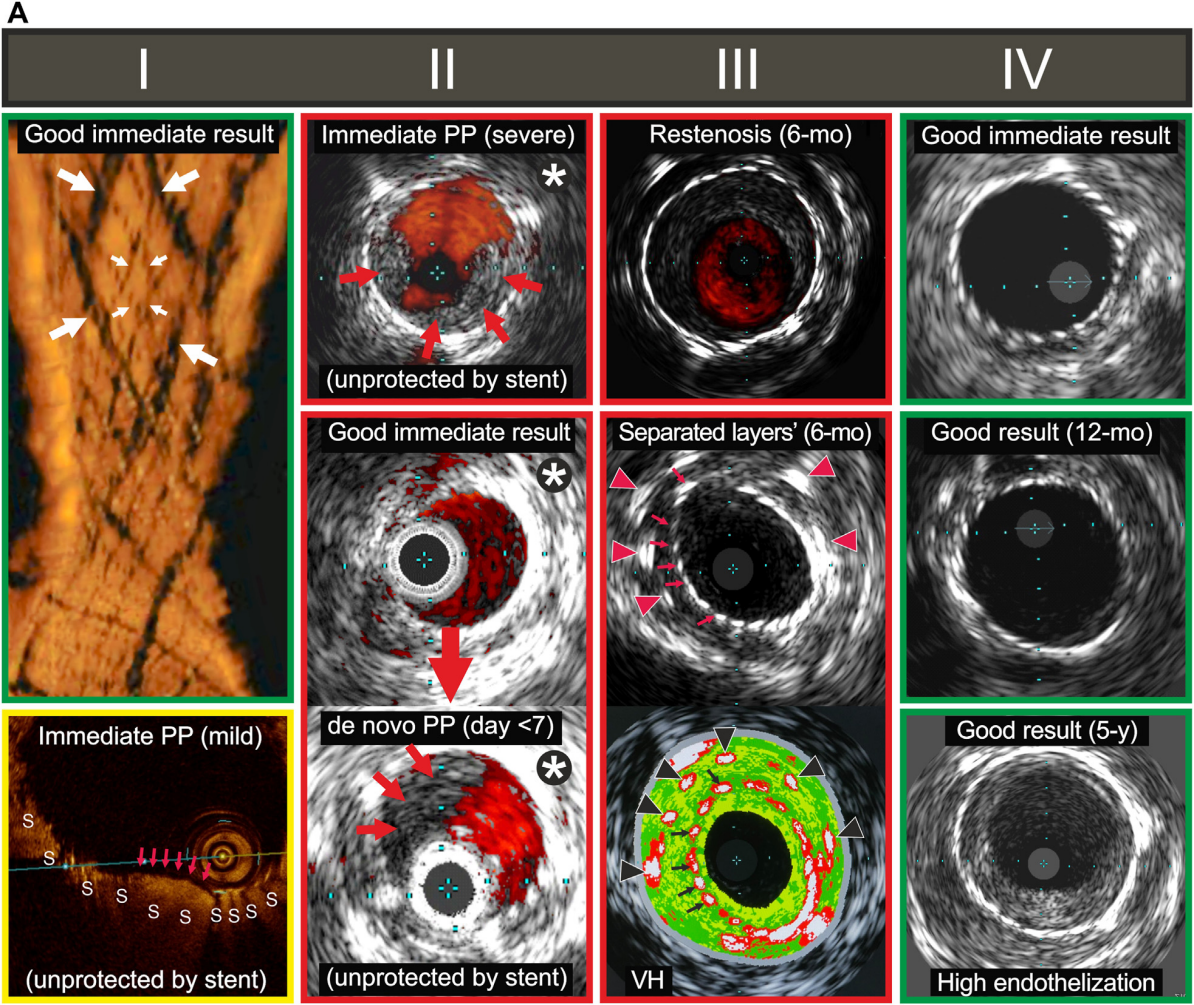

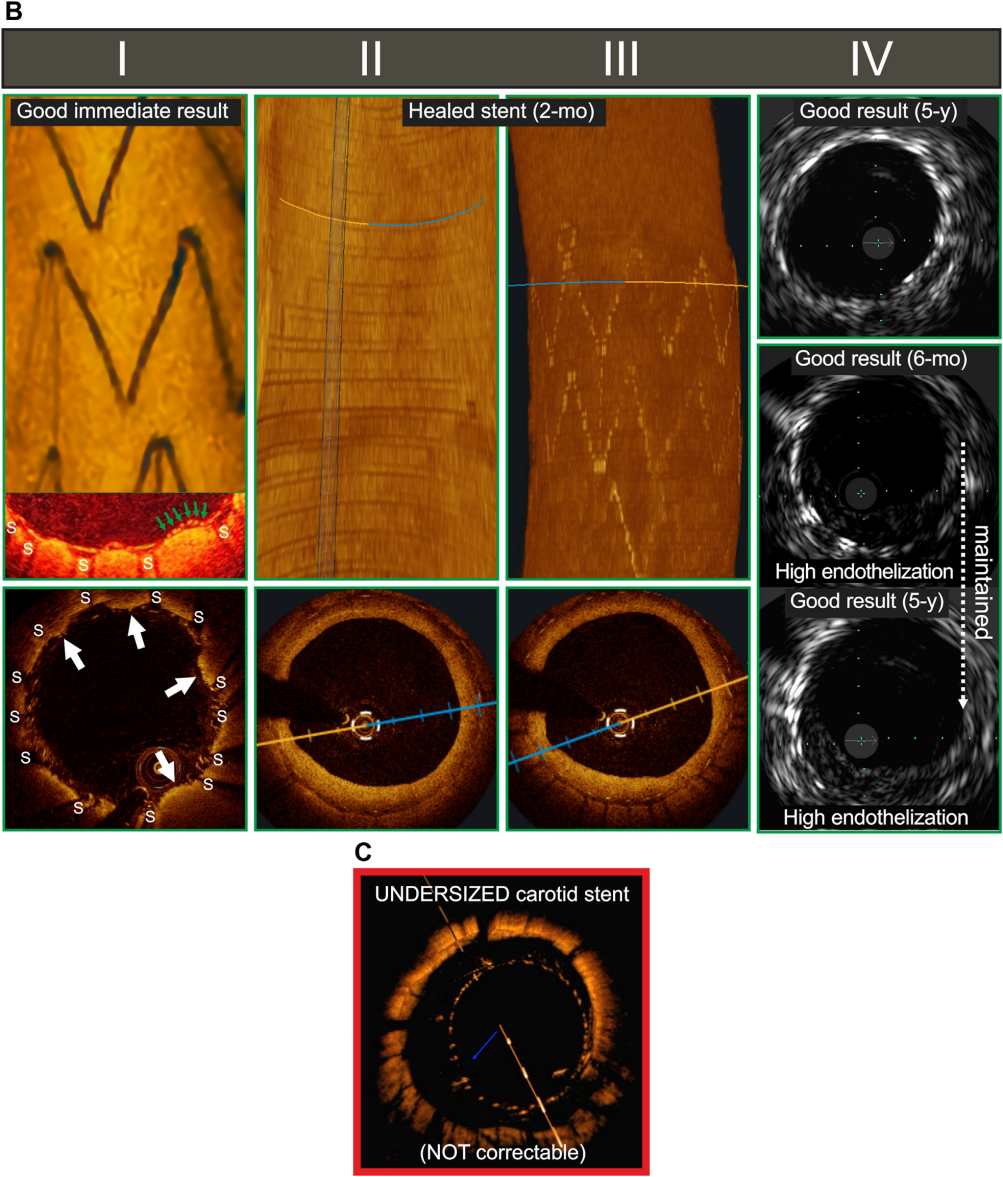
**Key examples of intravascular imaging of carotid “mesh” stents following implantation in humans as per indication for use.** (A) Immediate, early, mid-, and long-term in situ visualization of the dual metallic layer (double-braided) carotid “mesh” stent (Casper/Roadsaver). (Panel I) Optical coherence tomography (OCT) demonstration of a good immediate procedural result (top); longitudinal reconstruction “inside view” showing both the stent frame wires, thick arrows, and mesh wires positioned inside to the frame wires, thin arrows). Cross-sectional in the bottom shows immediate plaque prolapse (PP) of a mild magnitude (purple arrows; s – struts). Note that with the dual metallic layer design, intraluminal PP is not protected by the mesh because the protruding plaque penetrates through the mesh struts into the lumen. (Panel II) Intravascular ultrasound (IVUS, 20 Mz) visualization of immediate, severe PP (top) that requires addressing with another stent placement under cerebral embolic protection device (EPD). The middle image, obtained on procedure completion in another patient, shows lack of immediate PP in an underexpanded stent, with soft plaque presence outside the stent between ∼6 and 12 hours. However, several days later, severe PP is noted; this may be related to an increase in stent diameter with the self-expandable stent diameter “memory”^5^ (bottom image). The large magnitude of PP in this case requires trapping the embolic material—under EPD—with another stent to prevent spontaneous cerebral embolization. (Panel III) In-stent restenosis (ISR) at 6 months in a properly expanded stent (top; IVUS, area stenosis 62.3%, abnormal in-stent velocities on transcutaneous duplex Doppler imaging, considered as an indication for drug-eluting balloon angioplasty under EPD to prevent further ISR progression and mitigate the risk of neurologic symptoms). Mid and bottom image are, respectively, grayscale IVUS and virtual histology (VH) presentation of the consequence of separation of the stent frame and mesh that likely resulted from an attempt to optimize the stent lumen; arrowheads point to metallic struts of the external stent layer (frame) whereas arrows point to metallic struts of the inner stent layer (mesh); note that to minimize image obstruction not all the struts are labeled. There is also tissue formation inside to the detached mesh layer. For more details see text and observations reported in Ref.^6^ (Panel IV) IVUS presentations of a good immediate result (top), good result at 12 months (mid) and a good result at 5 years (bottom, high endothelization in absence of ISR). (B) Immediate, early, mid- and long-term in situ visualization of the MicroNET-covered carotid “mesh” stent (CGuard). (Panel I) OCT visualization of a good immediate procedural result (top; longitudinal reconstruction “inside view” with the stent frame struts in the front; an adaptable polyethylene terephthalate mesh wrap is outside to the metallic struts). Mid—a fragment of cross-sectional OCT frame (zoomed image with color arrangement displaying the MicroNET individual filaments, arrows, protecting plaque protrusion in between rarely positioned metallic struts, “s”). The bottom cross-sectional image demonstrates regions of plaque material crossing the luminal lines of rarely positioned metallic struts (arrows); note the mild PP magnitude due to its MicroNET protection—such a protected prolapse is highly unlikely to cause any postprocedural cerebral embolism.^4^ (Panel II and Panel III) A complete stent endothelization by OCT performed 2 months after implantation. Panel II is an “inside view” (3D reconstruction; the “rings” are imaging segment overlaps); bottom image demonstrates a complete endothelial layer covering the struts that are “rare” in this cross-section. Panel III (top) is a 3D reconstruction in the same patient/same OCT run (as Panel II), focused on strut visualization in the distal stent segment. The bottom image demonstrates a complete endothelial layer covering the struts that are more “dense” in this specific cross-section than in Panel II. Panel IV shows IVUS examples of a healed stent at 5 years (top) and stent healed with high endothelization between ∼2 hours and ∼9-hours at 6 months (mid) that is maintained at 5 years (bottom; high endothelization in absence of ISR). (C) Common carotid artery OCT cross-section demonstrating immediate consequence of selecting a too-small-diameter braided mesh-stent. Note (in this case—a complete) lack of stent apposition. This (1) cannot be corrected using endovascular approach, and (2) is associated with a significant risk of stent thrombosis and in-stent restenosis. Unless specified, images are from different patients. ∗Denotes images from the study by Myouchin et al^7^ published in this issue of *JSCAI*.

Casper/Roadsaver is a woven-wire, double metallic layer (each layer: braided) stent.^5^ The ∼180-μm thick woven wires (stent frame) cover a woven mesh of 42-μm wires. The outer layer resembles a braided single-layer carotid stent (Wallstent); the inner metallic layer forms pores of ∼0.15 to 0.20 mm^2^ (Figure 1A).^5^, ^6^, ^7^ The inner mesh is connected to the outer frame by ∼45-μm thick wires.^5^ The dual-braided structure of the Casper/Roadsaver stent results in poor adaptation to curves/lumen irregularities.^5^ The stent mean thickness, determined by 4 wire layers, is ∼1.7 mm (note ample space needing endothelization).^5^ This, taken together with a propensity for malapposition, may be prothrombogenic. Also, the final (postimplantation) stent length is difficult to predict (∼20%-30% elongation).^5^ Advantages are the low-profile (5F), flexibility on delivery, and feasibility to resheath after partial release.

The Gore Carotid Stent is composed of an open-cell (laser-cut) metallic frame and an (fixed on stent edges) ultra-thin Teflon outer layer with square-like perforations of ∼0.25 mm^2^.^8^ Depending on the diameter, the delivery profile is 5F or 6F.^8^

The CGuard is a widely open-cell (laser-cut) metallic frame (free cell area of ∼22 mm^2^; strut thickness ∼250 μm) that is wrapped by an outer, single-fiber knitted polyethylene terephthalate MicroNet adaptable sleeve (fiber thickness ∼25 μm; cell size ∼0.02-0.03 mm^2^; fixation on stent edges; delivery profile 6F).^9^ The current combined properties of (1) the most open-cell metallic stent frame, and thus highly conformable^9^ and (2) the smallest-cell antiembolic layer with a mesh pore size similar to embolic protection filters, result in a dense plaque coverage between the sparse struts (strut-to-strut distance up to ∼3.6 mm) and provide MicroNet protection in case of tissue prolapse beyond the strut line (Figure 1B).

After stent implantation, the risk of plaque prolapse (PP) and plaque-related embolism are linked to plaque characteristics^7^^,^^10^ that can be improved in the long term with aggressive lipid-lowering therapy^11^ and stent mechanical properties including scaffold density.^1^^,^^5^^,^^8^^,^^12^ The mesh aims to deliver permanent cerebral protection against plaque-related embolism.^1^ A recent large-scale meta-analysis (68,422 patients in 112 studies) demonstrated that overall second-generation stents perform substantially better than single-layer carotid stents at 30 days and 12 months.^8^ Nevertheless, clinically relevant differences were identified between specific stent designs, indicating lack of a uniform “mesh stent class” effect.^8^ Casper/Roadsaver reduced 30-day death/stroke/myocardial infarction (MI) vs single-layer stents (–2.78%, *P* = .02),^8^ and at 12 months, resulted in a significant reduction in ipsilateral stroke (–3.25%, *P* = .01) at the expense of an increased rate of in-stent restenosis (ISR) (+3.19%, *P* = .001).^8^ The Gore Carotid Stent showed outcomes similar to first-generation stents.^8^ CGuard reduced 30-day death/stroke/MI (–3.03%; *P* < .001), 12-month ipsilateral stroke (–3.13%; *P* = .01), and ISR (–3.63%; *P* < .01).^8^ Consistent with the meta-analytic model clinical event rates,^8^ the recent US Food and Drug Administration pivotal trial of the CGuard MicroNet-covered stent (C-GUARDIANS, NCT04900844, performed in a cohort of 316 high-risk patients including 41.8% diabetics) showed a 0.95% rate of death/stroke/MI at 30 days, and the 12-month composite end point of death/stroke/MI by 30 days plus any ipsilateral stroke at 31 days to 12 months <2%, with 1% any target lesion revascularization by 12 months.^13^

Optical coherence tomography evaluation demonstrated a 2-fold reduction in incidence of immediate post-procedural intraluminal PP with CGuard (where PP is limited to ∼100-150 μm depth and is protected by a MicroNet “parachute”; Figure 1B) vs Casper/Roadsaver (unprotected PP, Figure 1A).^6^ Clinical relevance of the Casper/Roadsaver unprotected PP likely depends on its magnitude.^6^^,^^7^^,^^14^ In the study by Matsumoto et al,^6^ Casper/Roadsaver PP was associated with documented cerebral embolism in 1 in 6 (17%) patients, but larger-scale data are needed to more firmly establish that association. Another mechanistic issue of the Casper/Roadsaver is the weak connection between the inner and outer metallic layers,^5^^,^^6^ translating into a nonnegligible (incidence >10%)^6^ risk of the layers’ separation. This may not only cause lumen loss with thrombus/tissue formation between the 2 separated layers but also stimulate thrombosis and restenosis intraluminal to the inner layer (eg, 12-month image in Figure 1A). Finally, the braided design—already when single-layered—is associated with a ∼3-fold increase in the risk of ISR vs other first-generation stents.^15^ It is thus not surprising to see a higher rate of ISR with the dual-layer double-braided design (ISR 7.86% at 12 months; 95% CI, 5.04%-10.68%).^8^

In the prospective study of 82 consecutive patients implanted with Casper/Roadsaver published the current issue of *JSCAI*, investigators from Japan^7^ demonstrate a new mechanistic finding that may further explain concerns with Casper/Roadsaver. Using ultrasound, Myouchin et al^7^ not only confirmed the phenomenon of immediate PP on CAS completion (incidence 2.2%) and a high ISR rate (6% at 12 months), but also demonstrated a significant de novo PP in the 2nd week post procedure in 3.4% patients. With routine use of transcutaneous rather than intravascular ultrasound in follow-up, which has ∼50% reduced resolution and anatomic/calcium-shadowing limitations as well as a single time point of assessment^7^ (additional evaluation at 14-21 days would be of interest), the true incidence of PP over time may be underestimated.

Recently, the European ROADSAVER Study in 1965 elective carotid stenosis patients, with embolic protection device (EPD) use in 63.8%, reported an overall 30-day stroke/death rate of 2.2% and 2.5% with <50% EPD use vs 2.0% with >80% EPD (18% proximal).^16^^,^^17^ This represents a 25% relative reduction in stroke/death associated with the use of EPD, consistent with data from large-scale registries^1^ emphasizing the important clinical role of routine EPD use in conjunction with antiembolic stents to provide optimal cerebral protection covering the procedural stages prior to the mesh-exerted antiembolic action. Note that the latter can occur only after the stent is fully implanted, thus the steps of lesion predilatation and the stent introduction through the lesion and its implantation remain unprotected by the mesh.^1^^,^^17^

Device evolution is part of the progress of medicine. Whether the dual metallic layer double-braided carotid stent^5^, ^6^, ^7^ will share the fate of the first marketed coronary drug-eluting stent or will remain in the carotid antiembolic stent armamentarium will largely depend on the pending clinical outcomes and restenosis rate in the rigorously controlled Casper/Roadsaver US Pivotal Study (CONFIDENCE trial, NCT02657707).

Finally, operators need to be cognizant that no stent is immune to user errors. Selection of an undersized stent (Figure 1C), suboptimal implantation techniques, including failure to address correctible malapposition and significant residual stenosis, or failure to adhere to instruction for use-determined anticoagulation and antiplatelet regimen can lead to poor results despite the best stent design.^18^
